# Genome-Wide Association Study in Immunocompetent Patients with Delayed Hypersensitivity to Sulfonamide Antimicrobials

**DOI:** 10.1371/journal.pone.0156000

**Published:** 2016-06-07

**Authors:** Jennifer M. Reinhart, Alison Motsinger-Reif, Allison Dickey, Steven Yale, Lauren A. Trepanier

**Affiliations:** 1 Department of Medical Sciences, School of Veterinary Medicine, University of Wisconsin-Madison, Madison, Wisconsin, United States of America; 2 Bioinformatics Research Center, Department of Statistics, North Carolina State University, Raleigh, North Carolina, United States of America; 3 Marshfield Clinic Research Foundation, Marshfield, Wisconsin, United States of America; Tianjin University, CHINA

## Abstract

**Background:**

Hypersensitivity (HS) reactions to sulfonamide antibiotics occur uncommonly, but with potentially severe clinical manifestations. A familial predisposition to sulfonamide HS is suspected, but robust predictive genetic risk factors have yet to be identified. Strongly linked genetic polymorphisms have been used clinically as screening tests for other HS reactions prior to administration of high-risk drugs.

**Objective:**

The purpose of this study was to evaluate for genetic risk of sulfonamide HS in the immunocompetent population using genome-wide association.

**Methods:**

Ninety-one patients with symptoms after trimethoprim-sulfamethoxazole (TMP-SMX) attributable to “probable” drug HS based on medical record review and the Naranjo Adverse Drug Reaction Probability Scale, and 184 age- and sex-matched patients who tolerated a therapeutic course of TMP-SMX, were included in a genome-wide association study using both common and rare variant techniques. Additionally, two subgroups of HS patients with a more refined clinical phenotype (fever and rash; or fever, rash and eosinophilia) were evaluated separately.

**Results:**

For the full dataset, no single nucleotide polymorphisms were suggestive of or reached genome-wide significance in the common variant analysis, nor was any genetic locus significant in the rare variant analysis. A single, possible gene locus association (*COL12A1*) was identified in the rare variant analysis for patients with both fever and rash, but the sample size was very small in this subgroup (n = 16), and this may be a false positive finding. No other significant associations were found for the subgroups.

**Conclusions:**

No convincing genetic risk factors for sulfonamide HS were identified in this population. These negative findings may be due to challenges in accurately confirming the phenotype in exanthematous drug eruptions, or to unidentified gene-environment interactions influencing sulfonamide HS.

## Introduction

Potentiated sulfonamides, such as sulfamethoxazole (SMX) in combination with trimethoprim (TMP), are effective antibiotics for the treatment of urinary infections, bronchitis, pneumonia, and methicillin-resistant *S*. *aureus* (MRSA) infections. [1–4] TMP-SMX is also the drug of choice for the prevention of opportunistic protozoal infections, such as toxoplasmosis and *Pneumocystis jiroveci* pneumonia, in immunocompromised patients, particularly in those with AIDS. [5–8]

Despite this broad-spectrum of activity, clinical use of TMP-SMX is limited by the development of idiosyncratic, delayed-type hypersensitivity (HS) reactions including fever and cutaneous drug eruptions and, less commonly, multi-organ dysfunction. [9] In fact, TMP-SMX is the leading cause of cutaneous hypersensitivity reactions, and the most common culprit in severe bullous skin eruptions such as Stevens-Johnson syndrome and toxic epidermal necrolysis, which carry up to 30% mortality. [10–14] Hypersensitivity reactions occur in approximately 3% of TMP-SMX-treated patients in the general population. [15,16] Both clinical reports and *ex vivo* drug challenge studies have suggested that this syndrome may be familial; thus a genetic basis for sulfonamide HS has been proposed. [17,18]

Pharmacogenetic risk for sulfonamide HS has only been evaluated in a limited number of studies. Unfortunately, most of these studies were in HIV-positive patients, for whom acquired, rather than genetic, risk is more likely. [19–23] Genetic studies have been sparse in immunocompetent patients and have focused predominately on enzymes responsible for SMX biotransformation or on glutathione pathways, which can neutralize reactive drug metabolites. Two reports suggested that “slow” acetylator *NAT2* genotypes were associated with sulfonamide HS. [24,25] However, in a larger study in our laboratory, we found no such association despite full resequencing of *NAT2* coding region. [26] In that same study we also investigated the sulfamethoxazole detoxification genes, *CYB5A* and *CYB5R3*, but polymorphism frequencies were low and were not significantly different between sulfonamide HS and tolerant patients. [26]

CYP2C9*2/*3 variant alleles have been associated with decreased production of the reactive, SMX-hydroxylamine (SMX-HA) metabolite in human liver microsomes; [27] however, allele frequency variation has not been investigated in immunocompetent patients with sulfonamide HS. [20] Further, an increased risk for cutaneous drug eruptions overall was reported for patients with *GSTM1* and *GSTT1* null genotypes, but this study population was mixed, with sulfonamide HS patients representing only 4/36 affected patients. [28] Finally, an association between an HLA-A30 haplotype and sulfonamide-induced skin eruptions was found in a Turkish population, but HLA genotypes have yet to be studied in other immunocompetent populations. [12]

Beyond these few candidate-gene studies, no other drug biotransformation, redox, or immunoregulatory genes have been evaluated for an association with sulfonamide HS. Genome-wide association studies (GWAS) have successfully identified genetic targets associated with a variety of other adverse drug reactions when a candidate gene approach has proved insufficient. [29–32] Therefore, the purpose of this study was to screen for genetic markers of sulfonamide HS in an immunocompetent population, compared to drug tolerant controls, using a GWAS design.

## Methods

### Patient Identification

Patients with delayed sulfonamide HS and patients tolerant of a course of sulfonamide antibiotics were identified through the Marshfield Clinic Research Foundation’s Personalized Medicine Research Project (PMRP), a cohort of over 20,000 patients who receive their medical care at the Marshfield Clinic, Marshfield, WI. These patients have previously provided informed consent for the use of their samples for biomedical research, linked to de-identified medical records data. [33] Medical records were searched electronically for a history of TMP-SMX administration or for a diagnosis of sulfonamide HS. Marshfield Clinic Research Foundation staff individually reviewed medical records, using a structured abstraction form to identify patients with sulfonamide HS. Each case was adjudicated to ensure consistency and accuracy. The abstraction form included the following eligibility criteria: (1) administration of TMP-SMX for at least 5 days prior to the adverse event; [9] (2) documentation of one or more new clinical signs after starting TMP-SMX, including fever with or without eosinophilia, skin rash, increases in liver enzyme activities, hyperbilirubinemia, blood dyscrasias (anemia, leukopenia or thrombocytopenia), pneumonitis, myocarditis, aseptic meningitis, polyarthritis, acute interstitial nephritis, toxic epidermal necrolysis, or Stevens-Johnson syndrome; [9] (3) lack of other clinical explanation for the adverse event; and (4) resolution of clinical signs with discontinuation of TMP-SMX alone. Patients with only gastrointestinal symptoms such as nausea, vomiting or diarrhea, [9] or with acute anaphylactoid reactions, [34,35] were excluded. Because some forms of immunosuppression, in particular AIDS, lead to a high acquired risk of SMX hypersensitivity, apparently independent of genotype, [20] immunocompromised patients, including those with HIV infection or undergoing immunosuppressive chemotherapy, were not eligible. These criteria together were designed to yield a score of 6 or more, or “probable” adverse reaction, using the Naranjo Adverse Drug Reaction scale. [36]

Control patients (“tolerant;” TOL) within the PMRP that were prescribed TMP-SMX were enrolled sequentially from medical records in random order, to provide a 2:1 match with the HS patients for race, gender, and decade of age at sulfonamide treatment. TOL patients must have been prescribed a course of TMP-SMX at a standard therapeutic daily dosage for at least 10 days, with adequate follow-up in the medical record to indicate that the drug was taken and tolerated without adverse event. Clinical and demographic variables, including body weight, dosage, duration of treatment, and reason for TMP-SMX prescription were also abstracted. Patient data were provided to the investigators in a de-identified format, and therefore the study protocol was reviewed and granted exemption from federal regulations by the UW Health Sciences human subjects Minimal Risk Institutional Review Board. One investigator (SY) from the PRMP did have direct access to patient information as a part of the validation process but the review board was aware of this at the time the exemption was granted.

### Genotyping, Quality Control, and Data Pre-analysis

DNA samples from 99 HS and 198 TOL patients from the PMRP were genotyped using one or more of the following platforms: HumanCoreExome, Illumina 660Hg18, Illumina 660Hg19, or Infinium (Illumina; San Diego, CA). Genotype-calls were performed in Genome Studio software (Illumina; San Diego, CA). For the HumanCoreExome, 660Hg18, and 660Hg19 platforms, the data was formatted for use in PLINK (http://pngu.mgh.harvard.edu/purcell/plink/). [37,38] The genotyped Infinium raw data files existed in the A/B format and the single nucleotide polymorphisms (SNPs) were converted to their corresponding alleles to match the PLINK format of the other platforms. SNPs in the Infinium dataset whose genotypes were denoted as either an indel (12,602) or had ambiguous strand designations (1,286) were removed. PLINK formatted files for the HumanCoreExome, 660Hg18, and 660Hg19 platforms are available on dbGaP, along with the raw text files for the Infinium platform.

For each platform, called data were filtered for quality control (QC) in PLINK. No patients were removed from the study due to low genotyping (efficiency < 90%). SNPs were excluded if the genotyping efficiency was < 98% or if the minor allele frequency (MAF) was < 0.01. [39] Trend tests for Hardy-Weinberg equilibrium were performed and variants with extreme deviations were also excluded from analyses. Q-Q plots were used to assess overall quality of the GWAS data, and the quality of any significant SNPs was evaluated manually after association analysis.

Data from the four platforms were combined to form a single dataset. However, few SNPs were shared among all four platforms. Therefore, to maximize the number of SNPs evaluated, only data from the HumanCoreExome and Infinium platforms were included in the final analysis. This resulted in the exclusion of 10 patients (4 HS, 6 TOL) who were only genotyped on the Illumina Hg660 systems. Imputation was performed on the combined dataset using SHAPEIT (https://mathgen.stats.ox.ac.uk/genetics_software/shapeit/shapeit.html#home) and IMPUTE2 (https://mathgen.stats.ox.ac.uk/impute/impute_v2.html) software. The EUR group of individuals (n = 379) in the 1000 Genomes Project reference panel (June 2014 release; 1000genomes.org) was used to phase the haplotypes because the majority of the patients in this study were Caucasian. 420 SNPs in the dataset were either missing in the reference panel or had strand incompatibilities and were removed. The number of genotyped SNPs used in the imputation was 226,516 and the resulting number of imputed SNPs that had an info score > 0.7 and that were used in the downstream analyses was 10,329,316.

Including relatives within the same association study can skew results, so the KING program (http://people.virginia.edu/~wc9c/KING/) was used to assess relatedness between subjects. Patients were removed if a relatedness of the 3^rd^ degree or less was identified, resulting in the exclusion of an additional 10 patients (3 HS, 7 TOL). Further population substructure was assessed using EIGENSOFT software (http://www.hsph.harvard.edu/alkes-price/software/) with a linkage disequilibrium pruned set (62,653) from the genotyped SNPs; principal component analysis identified two clear outliers (1 HS, 1 TOL) from the population, which were removed. The final dataset included 275 patients: 91 HS and 184 TOL.

### Common Variant Analysis

Multivariable logistic regression analysis was performed on the genotyped SNPs using PLINK and using SNP dosages for the imputed SNPs. 2-bit dummy-encoding was used to ensure that no genetic model assumptions were made. [39] The first four principle components, determined in EIGENSOFT, were included as covariates to minimize spurious associations due to underlying population substructure. The population was almost entirely Caucasian (99%), so race was not included as a covariate. Similarly, sex and age were not found to be significant covariates (coefficient p-value < 0.05), so were not included in the final model. Before calculating the genomic inflation factor (λ) for the combined genotyped and imputed common variant results, any SNPs with a MAF < 0.01 were removed along with any imputed SNPs that also existed in the genotyped set. This resulted in 226,505 genotyped SNPs and 8,066,491 imputed SNPs. λ was 1.03 and the p-values were adjusted to correct for it. The Q-Q plot is shown in S1 Fig. All SNPs were assessed in the final analysis, but those within candidate genes previously hypothesized to be involved in sulfonamide HS pathogenesis (Table 1) were of particular interest. Based on the number of comparisons including both the genotyped and the imputed SNPs, p ≤ 6.03 x 10^−7^ was considered suggestive of significance and p ≤ 6.03 x 10^−9^ was considered significant at the genome-wide level. The Bonferroni correction was used for multiple comparison corrections at an alpha level of 0.05.

**Table 1:** Candidate genes within the GWAS hypothesized to be involved in the pathogenesis of sulfonamide HS, and the mechanistic rationale for inclusion of each candidate gene.

Gene	Protein	Rationale
*CYP1A2*	Cytochrome P450, family 1, subfamily A, polypeptide 2	SMX biotransformation
*CYP2C8*	Cytochrome P450, family 2, subfamily C, polypeptide 8	SMX biotransformation
*CYP2D6*	Cytochrome P450, family 2, subfamily D, polypeptide 6	TMP biotransformation
*GCLC*	Glutamate-cysteine ligase, catalytic subunit	Glutathione pathways for reactive drug metabolites
*GCLM*	Glutamate-cysteine ligase, modifier subunit	Glutathione pathways
*GSS*	Glutathione synthetase	Glutathione pathways
*GSTM1*	Glutathione S-transferase mu 1	Glutathione pathways
*GSTP1*	Glutathione S-transferase pi 1	Glutathione pathways
*GSTT1*	Glutathione S-transferase tau 1	Glutathione pathways
*HLA-A*	Major histocompatibility complex, class I, A	Antigen presentation
*HLA-B*	Major histocompatibility complex, class I, B	Antigen presentation
*HLA-C*	Major histocompatibility complex, class I, C	Antigen presentation
*HLA-DQA1*	Major histocompatibility complex, class II, DQ alpha 1	Antigen presentation
*MARC1*	Mitochondrial amidoxime reducing component 1	SMX biotransformation
*MARC2*	Mitochondrial amidoxime reducing component 2	SMX biotransformation
*MPO*	Myeloperoxidase	SMX biotransformation
*NAT1*	N-acetyltransferase 1	SMX biotransformation
*NAT2*	N-acetyltransferase 2	SMX biotransformation

Assuming a multiplicative model of inheritance and a risk-allele frequency of 0.5, an *a priori* power calculation demonstrated that an available sample of 99 HS and 198 TOL patients would yield at least 80% power to detect a genetic risk model with a relative risk of ≥ 3.0, while maintaining an overall family-wise error rate of 10^−8^ (representing a correction at a genome-wide level).

### Rare Variant Analysis

The present study was not powered to detect a significant difference in allele frequencies for SNPs with low MAFs. [38] However, associations between low-MAF calls and various diseases have been previously identified, emphasizing the potential importance of these rare variants. [40–42] Therefore, we characterized the influence of rare variants using a combined multivariate and collapsing gene-level approach. [43,44] All SNPs with MAF < 0.03 within an individual gene were collapsed into a single covariate and each gene was included in a multivariate logistical regression model. 17,793 loci were included in this analysis and candidate genes were specifically evaluated. Several rare-variant burden tests (ind, prop, weight) and variance-component tests (skat, skat-o) were used including those that weighted for possible functional significance of individual SNPs. [45, 46] For each locus included in the analysis, p-values from the separate rare-variant tests were combined to yield a single p-value using Lancaster’s method. [47] Gene-level associations with model adjusted q-value ≤ 0.05 were considered significant.

### Subgroup Analyses

Because definitive diagnosis of sulfonamide HS is difficult and misclassification can occur, HS patients matching a more refined clinical phenotype were included in two separate subgroup analyses. The first subgroup (FEV-RASH) included patients exhibiting both a fever (body temperature > 98.8°F) and cutaneous drug eruption (rash) (n = 16). The second subgroup (FEV-RASH-EOS) included patients exhibiting fever, rash, and documented peripheral eosinophilia (eosinophils > 0.5 M/l) on complete blood count (n = 8). Each subgroup was compared with the full control group (n = 184) in both a common and a rare variant analysis. The genotyped (226,516) and imputed SNPs (10,329,316) that were used as input for the full set common and rare variant analyses were also used as input for the subgroup analyses. After quality filtering the common variant analysis results, 226,444 genotyped and 8,077,026 imputed SNPs remained for subgroup FEV-RASH. λ was 0.87 and the resulting Q-Q plot can be seen in S2 Fig. 17,766 loci were included in the rare variant analysis. For subgroup FEV-RASH-EOS, 226,415 genotyped and 8,078,605 imputed SNPs remained after the common variant analysis results were quality filtered. λ was 0.68 and the resulting Q-Q plot can be seen in S3 Fig. 17,778 loci were included in the rare variant analysis in this subgroup. Candidate genes were specifically evaluated in both the common and rare variant analyses.

## Results

### Patient Population

**Fig 1:**
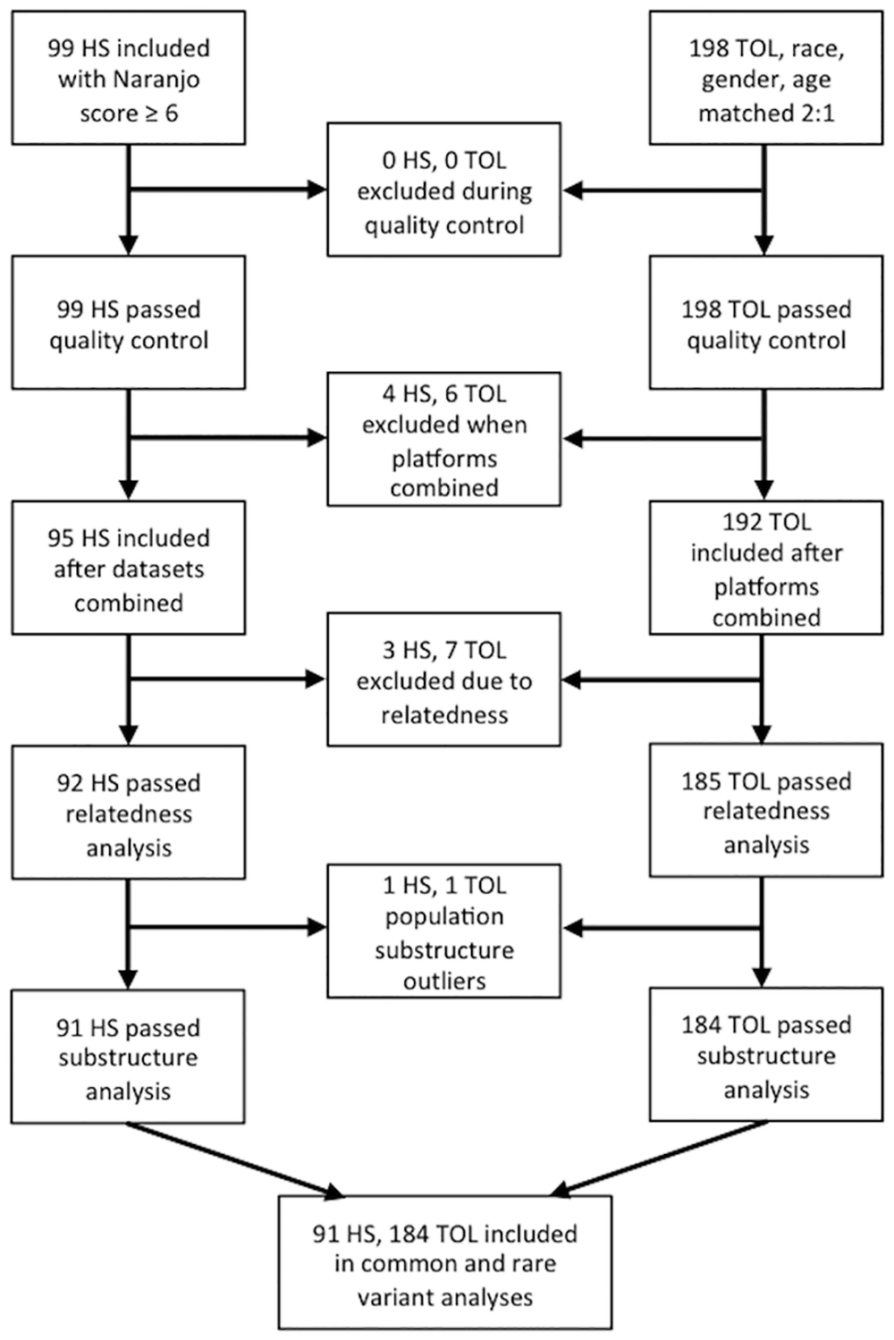
Inclusion and exclusion of recruited subjects.

Ninety-one HS and 184 TOL patients were included in the final analysis. Inclusion and exclusion of subjects are represented in Fig 1. Patient demographics are summarized in Table 2. HS patients were predominantly Caucasian (98%) and female (82%), with a median age of sulfonamide treatment of 38 years (range 1–80). Most of the HS patients had exanthematous (95%) or bullous (1%) drug eruptions. Other clinical manifestations included fever (20%), eosinophilia (17%), thrombocytopenia (11%), neutropenia (5%), anemia (4%), or elevated liver enzymes/hyperbilirubinemia (3%). Matched TOL patients were predominantly Caucasian (99%) and female (83%), with a median age of sulfonamide treatment of 41 years (range 8–88).

**Table 2:** Demographic information for sulfonamide hypersensitive (HS) and drug-tolerant (TOL) patients. Continuous data are presented as mean ± standard deviation (range). FEV-RASH = hypersensitive subgroup with fever and rash; FEV-RASH-EOS = hypersensitive subgroup with fever, rash, and eosinophilia. Diagnosis represents the underlying rationale for TMP/SMX prescription. (Note: body weight and therefore total daily dose in mg/kg were not available for 58 patients.)

	HS (n = 91)	FEV-RASH (n = 16) Subgroup of HS	FEV-RASH-EOS (n = 8) Subgroup of HS	TOL (n = 184)
Age at Administration (yr)	40.0 ± 17.2 (1.1–80.4)	40.8 ± 18.7 (1.1–80.4)	46.5 ± 15.0 (24.3–70.1)	41.1 ± 17.3 (8.5–87.7)
Body weight (kg)	77.0 ± 19.2 (13–124)	84.0 ± 16.0 (55–105)	87.0 ± 17.0 (61–97)	85.3 ± 21.4 (27.2–150.0)
Total Daily Dose (mg/kg)	25.7 ± 6.2 (10.3–37.5)	22.9 ± 6.7 (10.3–35.1)	23.0 ± 5.6 (19.8–31.3)	23.5 ± 6.2 (7.0–40.6)
***GENDER***				
Female	75	10	4	153
Male	16	6	4	31
***RACE***				
Caucasian	89	15	7	183
Native American	2	1	1	1
***DIAGNOSIS***				
Urinary Tract Infection	32	5	3	41
Respiratory Tract Infection	55	9	4	101
Other Soft Tissue Infection	2	1	1	38
Unknown/Multiple	2	1	0	4

### Common Variant Analysis

**Fig 2:**
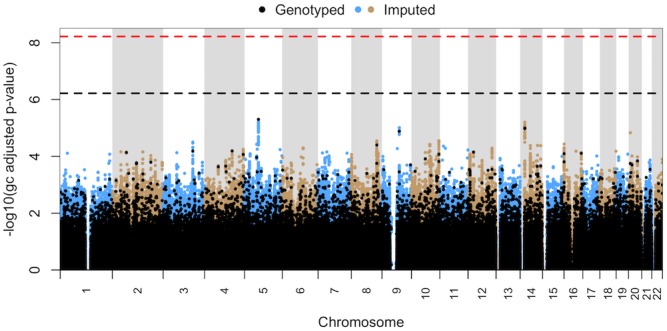
Manhattan plot for common variant analysis of full set of sulfonamide hypersensitive (HS) vs. drug tolerant (TOL) patients. No SNPs were suggestive of (p ≤ 6.03 x 10^−7^) or reached genome-wide significance (p ≤ 6.03 x 10^−9^).

The Manhattan plot of the full dataset is shown in Fig 2. No SNP was suggestive of, or reached, genome-wide significance. Similarly, no SNP in any candidate gene reached significance. The SNP with the lowest p-value was rs160978 (p = 4.98 x 10^−6^) located in an intergenic region of chromosome 5.

### Rare Variant Analysis

Of the 17,793 loci included in this gene-level analysis, there were no statistically significant differences between HS and TOL patients in the full data set. In particular, there were no significant differences for any of the candidate genes. The gene with the lowest q-value was *FNBP1* (q = 0.2558), located on chromosome 9, which codes for formin binding protein 1.

### Subgroup Analyses

**Fig 3:**
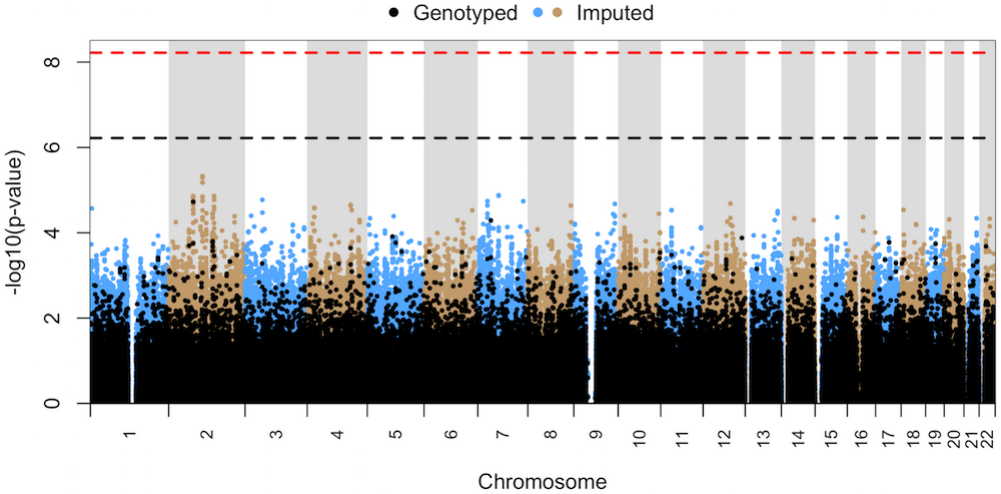
Manhattan plot for common variant analysis of the HS subgroup with rash and fever (FEV-RASH) vs. all TOL patients. No SNPs were suggestive of (p ≤ 6.02 x 10^−7^) or reached genome-wide significance (p ≤ 6.02 x 10^−9^).

The 16 patients included in subgroup FEV-RASH were predominantly Caucasian (15/16) and female (10/16) with a median age of 42 years (range 1–70). In addition to fever and rash, 7 had blood dyscrasias (neutropenia, thrombocytopenia, and/or anemia) and 1 had bullous skin eruptions. In the common variant analysis, no SNP was suggestive of or reached genome-wide significance (Fig 3). However, in the rare variant analysis, there was a statistically significant difference between the TOL and FEV-RASH groups for the gene *COL12A1* (q = 0.0167) on chromosome 6, which codes for type XII α1 collagen.

**Fig 4:**
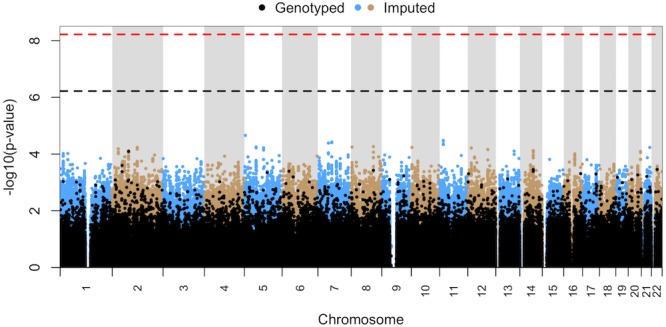
Manhattan plot for common variant analysis of HS subgroup with fever, rash and eosinophilia (FEV-RASH-EOS) vs. all TOL patients. No SNPs were suggestive of (p ≤ 6.02 x 10^−7^) or reached genome-wide significance (p ≤ 6.02 x 10^−9^).

The 8 patients in subgroup FEV-RASH-EOS were predominantly Caucasian (7/8) with a median age of 48 years (range 24–70). Males (4/8) and females (4/8) were equally distributed. Six of these patients had blood dyscrasias and 1 exhibited bullous skin eruptions. In the common variant analysis, no SNP was suggestive of, or reached, genome-wide significance (Fig 4). In the rare variant analysis, there were no significant differences between TOL and FEV-RASH-EOS patients for any locus, including *COL12A1*.

No SNP or locus within the candidate gene set was significant for either subgroup in either the common or rare variant analysis. The top 100,000 SNP associations in the common variant analyses for the full data set and each subgroup are reported in S1 Zip. All associations in the rare variant analyses for the full data set and each subgroup are reported in S2 Zip.

## Discussion

In the past 15 years, many pharmacogenetic studies have used novel, genome-wide techniques to identify previously unknown genetic risk factors for drug HS reactions. [29–32] Many of the strongest associations have been found for the HLA loci, but other metabolic, transporter, and drug-target genes have also been implicated. [29–32,48–55] Such associations are particularly important to the rapidly growing field of personalized medicine; in fact, the FDA now recommends prospective genotyping for some of these implicated drugs. [56] Although a genetic basis for sulfonamide HS has long been suspected, little work has been done in immunocompetent patients and none at the genomic level. [9] Therefore, the aim of this study was to identify possible genetic risk factors for sulfonamide HS in the general population using a GWAS approach. Unfortunately, the present study did not demonstrate any genetic associations for sulfonamide HS. These negative findings could result from insufficient study sensitivity, inadequate clinical phenotyping, or represent a true lack of high impact genetic effects for this drug HS syndrome in this population.

The power of a GWAS lies in its ability to simultaneous assess millions of variants across tens of thousands of genes. This provides superior sensitivity over a traditional candidate gene approach in which only one or few genes are investigated. Candidate studies also require an index of suspicion on the part of the investigators that a gene may be mechanistically important to the trait of interest, whereas a genome-wide approach is free from such investigator bias. However, the ability of any study to detect significant differences between groups relies on its sample size and, because a GWAS involves millions of comparisons, large sample sizes are usually required to maintain adequate study sensitivity. The present study was powered to detect a 3-fold increase in relative risk. Pharmacogenetic studies of drug outcomes are typically powered to detect only variants with large genetic effects, because polymorphisms with small effects are unlikely to be adequately predictive to impact clinical decision making. [57] Although the present study was sufficiently powered to detect a relative risk of ≥ 3.0, genotyping more HS and TOL patients would have increased sensitivity and may have allowed us to detect significant risk of lesser magnitude.

Because it is likely that not all of the SNPs are independent, our significance cutoff value of 6.03 x 10^−9^ may have been overly conservative. If the significance cutoff value were based only on the genotyped SNPs, the p-value would increase from 6.03 x 10^−9^ to 2.21 x 10^−7^. Since the smallest p-value found in this study was 4.98 x 10^−6^, the study conclusions remain the same whether the genotyped SNPs alone, or the genotyped and the imputed SNPs, are used in calculating the significance cutoff value.

The rare variant analysis used in this study accounted for the genetic effects of low MAF SNPs that would have been missed in a traditional GWAS approach. However, this technique does not detect genetic interactions. Development of methods to detect interactions at the SNP, gene, and pathway level is an ongoing area of bioinformatics research. [58–60] Study sensitivity may also have been improved had we used a different modality for genotyping. This GWAS was performed using SNP arrays, which included hundreds of thousands of known variants. Combined with imputation, several million genetic markers were used in this study. However, next-generation, whole-genome sequencing exponentially increases the number of possible SNPs included and also assesses non-SNP variants (e.g. indels, inversions, transpositions). Whole-genome sequencing would also allow direct identification of the causative variant, rather than relying on linkage disequilibrium to identify an area of the genome, which then must be re-sequenced.

Our negative findings emphasize the difficulties in studying patients with a diagnosis of drug hypersensitivity as manifested by exanthematous rash, which can have other etiologies that could be misdiagnosed as a drug reaction. We attempted to minimize this by direct medical record review and use of a validated adverse drug reaction scale. Despite these measures, errors and misinterpretation of medical records can still occur. For example, upon secondary review of the cases, it was discovered that one TOL subject failed to complete the minimum 10-day course of TMP/SMX due to GI upset. Given that our analyses did not reveal any significant findings, this oversight is unlikely to affect our results, but it does highlight the difficulties in reviewing medical record information in retrospective studies. Furthermore, drug rechallenge remains the gold standard for confirming adverse drug reactions, [36,61] and this was not performed in most patients in our HS group for ethical reasons. In addition, only banked DNA samples were available for most patients, so we were unable to include other potential measures of causality, such as the lymphocyte transformation test or the *in vitro* cytotoxicity assay. [16,62,63] These additional biomarkers may have refined our HS population and possibly provided a more clinically uniform phenotype to study.

Because of concerns about phenotype, we also analyzed a subgroup of patients with fever and rash (FEV-RASH) and a subgroup of patients with characteristics of the DRESS (drug reaction with eosinophilia and systemic signs) syndrome (FEV-RASH-EOS). [64] A single gene, *COL12A1*, was significant for the FEV-RASH group in the rare variant analysis. This gene encodes for type XII collagen, a regulator protein mediating the interaction between type I collagen and the extracellular matrix. [65] Although *COL12A1* has been associated with certain familial myopathies, [66] it is not known to be an immunoreactive protein, and there does not seem to be a logical role for a collagen protein in our current understanding of sulfonamide HS pathogenesis. Thus, this modest association was likely due to a Type I error, particularly since the subgroup analyses were underpowered and at risk for sample size bias.

The lack of identifiable genetic associations for sulfonamide HS could be due to the methodical concerns discussed above, but could also represent a true lack of genetic effect for this syndrome. Although a few studies do support a familial inheritance pattern, [17,18] most of the evidence is anecdotal. Recent methods developments have allowed for an estimate of the overall phenotypic variance from GWAS data, but the current study was underpowered to get a reliable estimate. [67] Such estimates are often a challenge in pharmacogenomics studies. [68] With a potentially heritable trait, a genome-wide linkage study among members of an affected family appears an attractive option to minimize enrollment and maximize results. However, it is unusual for all family members to have been treated with the same drug. Furthermore, ethical considerations preclude prospective phenotyping by drug challenge in persons who may have a hereditary predisposition to sulfonamide HS.

Epigenetic and environmental factors may also play a role in the development of sulfonamide HS reactions. For example, patients infected with HIV have an incidence of sulfonamide HS of 20–57% compared to 3% in the general population. [69–72] This significant overrepresentation, along with the fact that risk for a reaction increases with declining immune status in AIDS patients, [70,73–76] implies that HIV is an acquired risk factor for sulfonamide HS. Several cases of TMP-SMX-induced HS reactions have also been reported during recrudescence of human herpesvirus-6 (HHV6) infection. [77–79] Although a cause and effect relationship has not been established, it has been suggested that HHV6 reactivation induces pro-inflammatory cytokines, which might predispose to a cell-mediated immune response, constituting another possible acquired risk factor for sulfonamide HS even in the immunocompetent population. [78,79]

In conclusion, we did not identify any convincing genetic associations for sulfonamide HS, manifested primarily as delayed onset of exanthematous drug eruption with or without fever and eosinophilia, in immunocompetent patients in our Caucasian-American population. These negative findings highlight the need for careful population phenotyping. Clinical use of assays for drug specific T cells or *in vitro* cytotoxicity may be useful, but new biomarkers for confirming HS reactions are also needed. Large, prospective, multicenter studies would allow for real-time evaluation of phenotype rather than relying on medical record adjudication. Additional studies are currently underway in our laboratory to further investigate the immunopathogenesis of sulfonamide HS.

## Supporting Information

S1 Fig Q-Q plot for common variant analysis of full set of sulfonamide hypersensitive (HS) vs. drug tolerant (TOL) patients (λ = 1.03). (TIF)

S2 Fig Q-Q plot for common variant analysis of HS subgroup with rash and fever (FEV-RASH) vs. all TOL patients (λ = 0.87). (TIF)

S3 Fig Q-Q plot for common variant analysis of HS subgroup with rash, fever, and eosinophilia (FEV-RASH-EOS) vs. all TOL patients (λ = 0.68). (TIF)

S1 Zip Text files including the top 100,000 SNP associations for the common variant analyses for the full data set as well as both subgroups (FEV-RASH and FEV-RASH-EOS). (ZIP)

S2 Zip Text files including all associations for the rare variant analyses for the full data set as well as both subgroups (FEV-RASH and FEV-RASH-EOS). (ZIP)
