# Supplementary figures and images for: Genome-Wide Association Study in Immunocompetent Patients with Delayed Hypersensitivity to Sulfonamide Antimicrobials
Source: PLoS One. 2016 Jun 7;11(6):e0156000. doi: 10.1371/journal.pone.0156000 (PMC4896425; doi:10.1371/journal.pone.0156000)

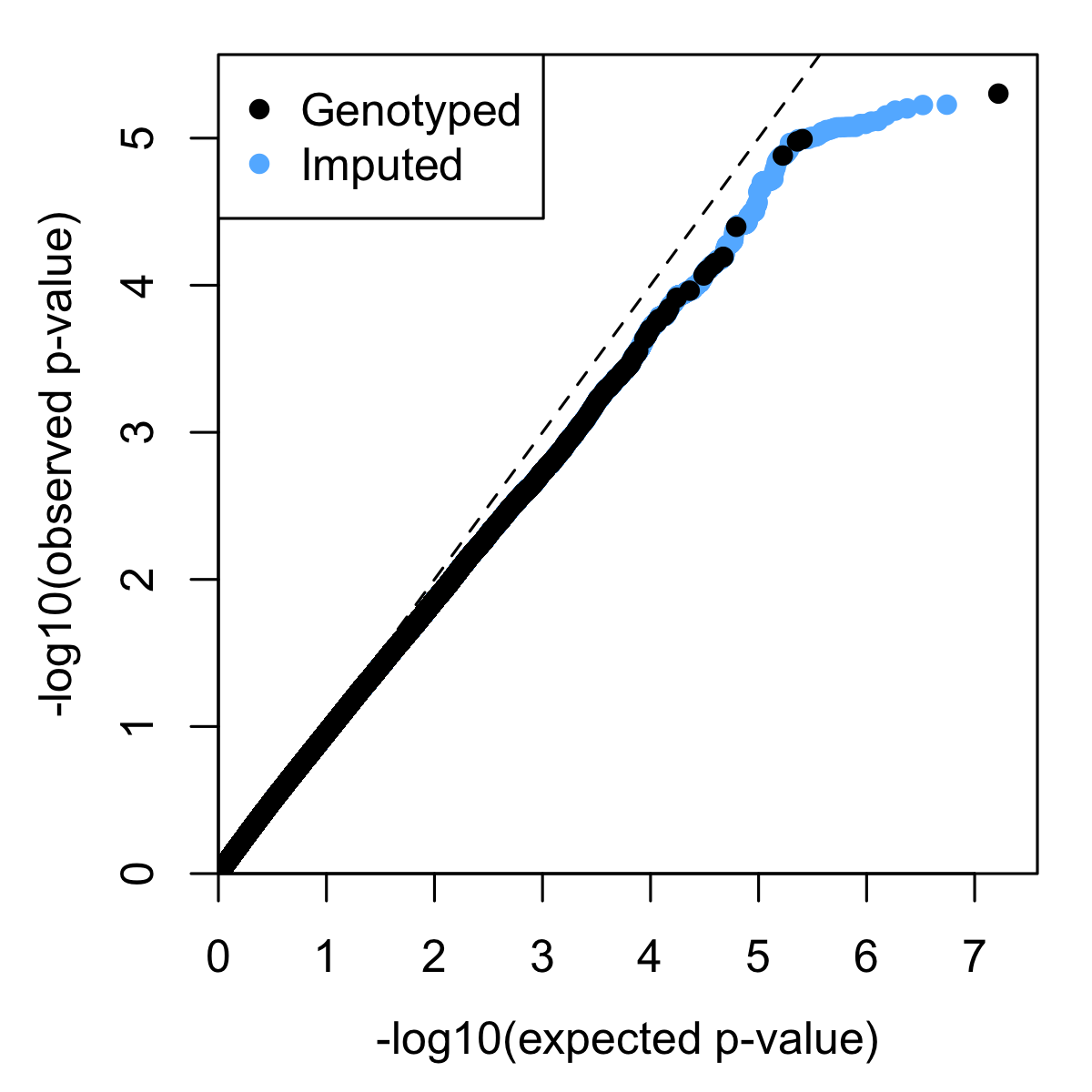

Supplement: S1 Fig — (TIF) [file pone.0156000.s001.tif]

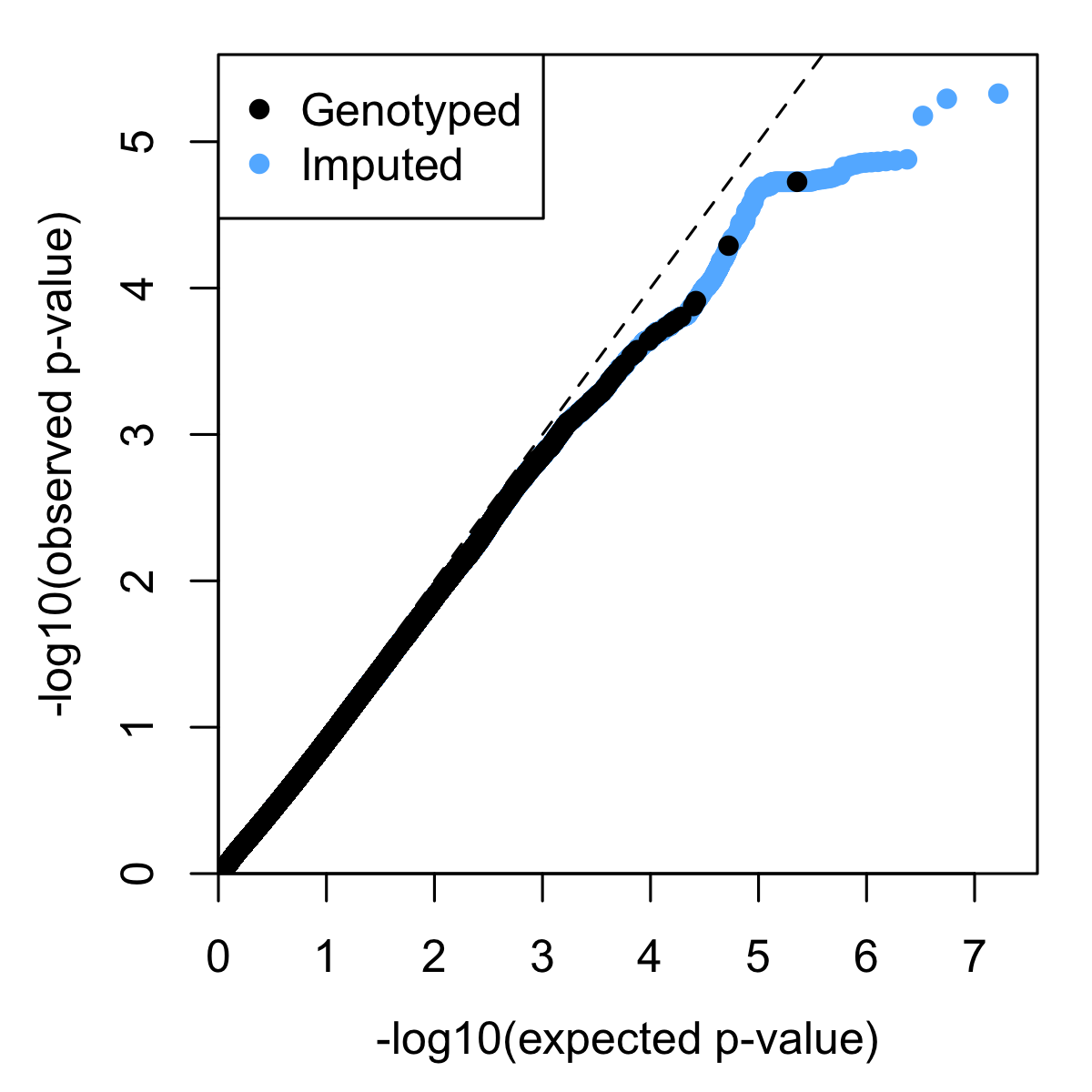

Supplement: S2 Fig — (TIF) [file pone.0156000.s002.tif]

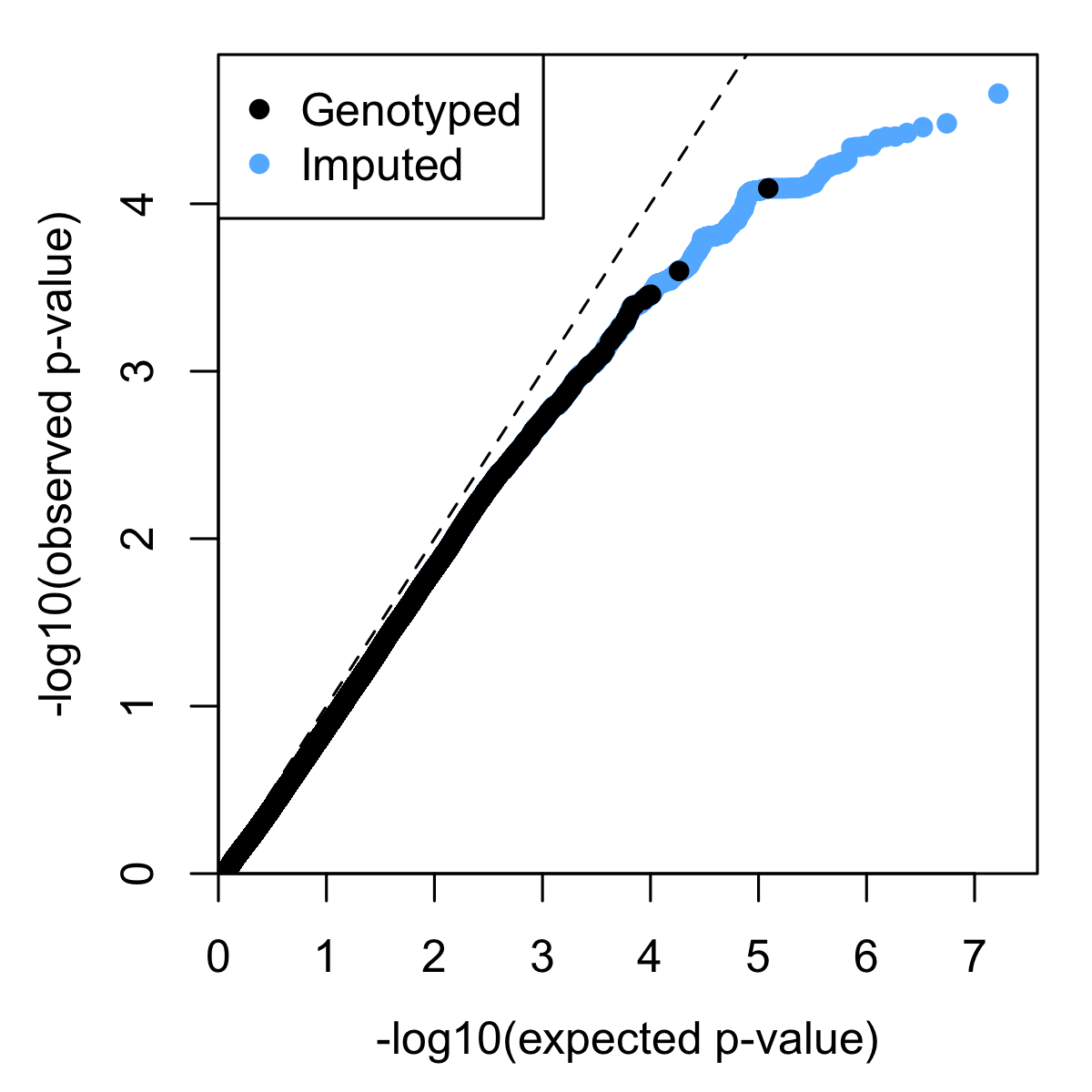

Supplement: S3 Fig — (TIF) [file pone.0156000.s003.tif]
